# Transcriptomic profile analysis of the halophyte *Suaeda rigida* response and tolerance under NaCl stress

**DOI:** 10.1038/s41598-020-71529-2

**Published:** 2020-09-16

**Authors:** Zhan-Jiang Han, Yang Sun, Min Zhang, Jun-Tuan Zhai

**Affiliations:** 1 College of Life Sciences, Tarim University, Alar, 843300 Xinjiang China; 2 Xinjiang Production & Construction Corps Key Laboratory of Protection and Utilization of Biological Resources in Tarim Basin, Alar, 843300 Xinjiang China

**Keywords:** Genome informatics, Plant molecular biology, Plant stress responses

## Abstract

*Suaeda rigida* is a lignified, true haplotype that predominantly grows in the Tarim basin, China. It has significant economic and ecological value. Herein, with aim to determine the genes associated with salt tolerance, transcriptome sequencing was performed on its stem, leaves and root over three set NaCl gradients regimens at treatment intervals of 3 h and 5 days. From our findings, we identified 829,095 unigenes, with 331,394 being successfully matched to at least one annotation database. In roots, under 3 h treatment, no up-regulated DEGs were identified in 100 and 500 mM NaCl treated samples. Under 5 days treatment, 97, 60 and 242 up-regulated DEGs were identified in 100, 300, 500 mM NaCl treated samples, respectively. We identified 50, 22 and 255 down-regulated DEGs in 100, 300, 500 mM NaCl treated samples, respectively. GO biological process enrichment analysis established that down-regulated DEGs were associated with nitrogen compound transport, organic substance transport and intracellular protein transport while the up-regulated genes were enriched in cell wall biogenesis, such as plant-type cell wall biogenesis, cell wall assembly, extracellular matrix organization and plant-type cell wall organization. These findings provide valuable knowledge on genes associated with salt tolerance of *Suaeda rigida*, and can be applied in other downstream haplotype studies.

## Introduction

The growth and development of plants is highly affected by several abiotic stress factors. These factors may include salinity, temperature, drought and heavy metals. Of these many abiotic factors, salinity is a significant environmental factor that affects plant productivity^1^. Globally, it is estimated that salinity affects almost 1 billion hectares of land^2^, a situation that has continued to be more critical due to the rising changes in climatic conditions^3^.

Congruently, plants possess various intricate adaptive responses to these stress factors^4^. In plants with ability to survive high salinity (halophytes), their response and adaptation to high saline levels typically relies on succulence buildup in their cells and tissues^5–9^. These high salt tolerance plants include representatives such as *Sonneratia acida*,* Pentatropis sianshoides*,* S. nudiflra*,* S. nudiflra*,* Limnitzera racemosa*,* S. maritima*,* Salvadora persica*,* S. salsa* and *S. rigida*^10–13^. These halophyte plants have the ability to compartmentalize the disproportionate Na+ and Cl− ions that are present in the cell vacuole, a mechanism that aids them in reducing the cells’ water potential and thereafter enhance their water absorbance ability from the saline soil, thus providing optimum ion concentration conditions in the cell cytoplasm for enzymatic and other biological activities^14–16^.

A previous study had shown that even though the *Arabidopsis thaliana* has a weak salt tolerance, and its response mechanisms is centered on the action of SALT OVERLY SENSITIVE3 [SOS3], a sensor, and SOS2, a protein kinase. These two protein complexes are implicated in detecting and responding to Na+ influx^17–20^, involving activation of SOS1, a plasma membrane-located sodium/proton antiporter. The SOS1 activation mediates the transfer and redistribution of the Na+ ions all over the plant^21–23^. Equally, SOS complex has been found to exist in other plant species, hence it may be a general feature of plants^24^. Equally, a study on the effect of salinity on *Suaeda fruticosa* linked its salt tolerance to its capability to uptake K+ so as to retain a higher K+/Na+ balance in the shoots^25^. Further, halophytes have mechanisms capable of regulating several biological pathways like signal transduction, energy metabolism and photosynthesis^26^.Other studies on plants’ salt and drought tolerance have also recognized several inducible stress regulators, with up-regulation of catalase, aquaporins, peroxidase and proline accumulation^27^. Moreover, plant abscisic acid (ABA) signaling pathways have been shown to be stimulated via stress factors^28^. The Sodium and chloride build-up in plant’s cytoplasm leads to cell cytotoxicity induced by accumulation of reactive oxygen species (ROS)^29^; a situation that can cause protein and lipid degradation, which may disrupts the cell functions^30^.

*Suaeda rigida* (HW Kung et GL Chu) is a lignified plant under genus *Polygonaceae*. It is a true haplotype plant that is endemic in the Tarim basin in Xinjiang, North west China. It is a nutrient-rich wild vegetable that is also an excellent local forage resource with important economic and ecological value in the region^31^. Notably, nearly one-third of the cultivated land in Xinjiang province is salinized, and the development and utilization of salt-tolerant plants form a key focal point in plant research in this region. Overall, study of plants’ salt tolerance mechanisms and the mining of related genes have important theoretical and practical significance in the development and utilization of excellent germplasm resources, enhancement of crop stress resistance and improvement of agricultural production. However, presently there is a lack of detailed knowledge on the saline stress and associated signaling pathways of *Suaeda rigida* plant. Therefore this study was aimed at applying transcriptomic sequencing in identifying and analyzing the putative genes implicated in salt response and tolerance mechanisms of *Suaeda rigida* plants over a set gradient of NaCl treatment regimens.

## Results

### Sequencing summary for all the libraries

A total of 343.88 GB of raw reads were obtained from the 42 libraries of the roots, leafs and stem samples (Supplementary Data 1). The average sequencing error rate was 0.029% while the average GC content was 42.76%. The Q20 and Q30 data ratio of sequencing quality was 97.10% and 92.35%, respectively (Supplementary Data 1). Since *S. rigida* does not have a reference genome yet, Trinity software was utilized to de novo assemble the obtained clean reads. In total, 2,212 million high-quality clean reads were de novo assembled into 829,095 transcripts with an N50 length 1453 bp and an N90 length of 269 bp (Table 1).

**Table 1:** Summary of transcriptome assembly.

Category	Number				Total	Mean length (bp)	N50 (bp)	N90 (bp)
	200–500 bp	500–1 kbp	1 k–2 kbp	> 2 kbp				
Transcripts	518,152	140,336	97,377	73,230	829,095	359	1,453	269
Unigenes	135,845	127,207	96,747	73,154	432,953	759	1,885	531

### Expression analysis, functional annotation, gene ontology assignments and analysis

**Figure 1:**
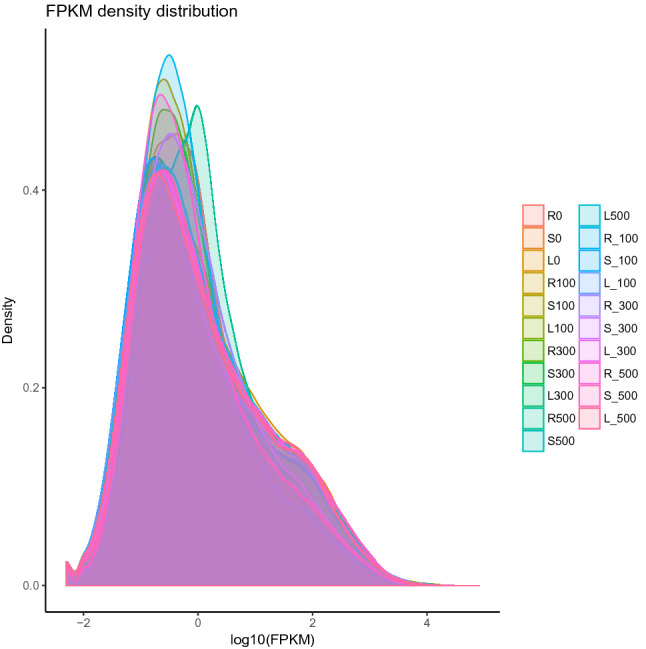
Comparison of gene expression levels under different experimental conditions.

After mapping of all the filtered reads to the de novo assembled contigs, we obtained all the expression data for them in all libraries. Thereafter, DESeq was utilized in identifying the differentially expressed genes. Comparison of gene expression analysis for each different experimental condition are as illustrated in Fig. 1.

Gene function annotation was attained by searching the obtained transcripts against the Nr, Nt, KO, Swiss-Prot, Pfam, GO and KOG databases. In sum, 331,394 unigenes successfully matched with at least one database, with 253,490 (58.54%), 241,135 (55.69%), 116,108 (26.81%), 224,239 (51.79%), 219,455 (50.68%), 221,659 (51.19%), and 113,379 (26.18%) annotated transcripts showing a significant hit against the Nr, Nt, KO, Swiss-Prot, Pfam, GO and KOG, respectively (Table 2).

**Table 2:** Gene annotation statistics.

	Number of genes	Percentage (%)
Annotated in NR	253,490	58.54
Annotated in NT	241,135	55.69
Annotated in KO	116,108	26.81
Annotated in SwissProt	224,239	51.79
Annotated in PFAM	219,455	50.68
Annotated in GO	221,659	51.19
Annotated in KOG	113,379	26.18
Annotated in all databases	57,824	13.35
Annotated in at least one database	331,394	76.54
Total unigenes	432,953	100

**Figure 2:**
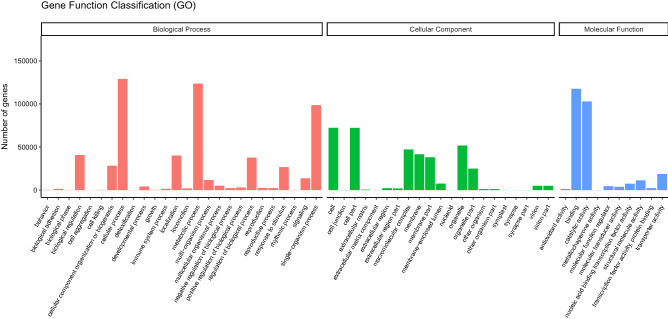
GO classification. The figure comprises of biological processes, cellular component and molecular function.

GO categorization established that cellular process, metabolic process and single-organism processes were the highly enriched biological process whereas, the genes associated with cell, cell part and organelle as the most significant among the cellular component terms. Three molecular function GO terms; binding, catalytic activity and transporter activity were significantly enriched (Fig. 2).

**Figure 3:**
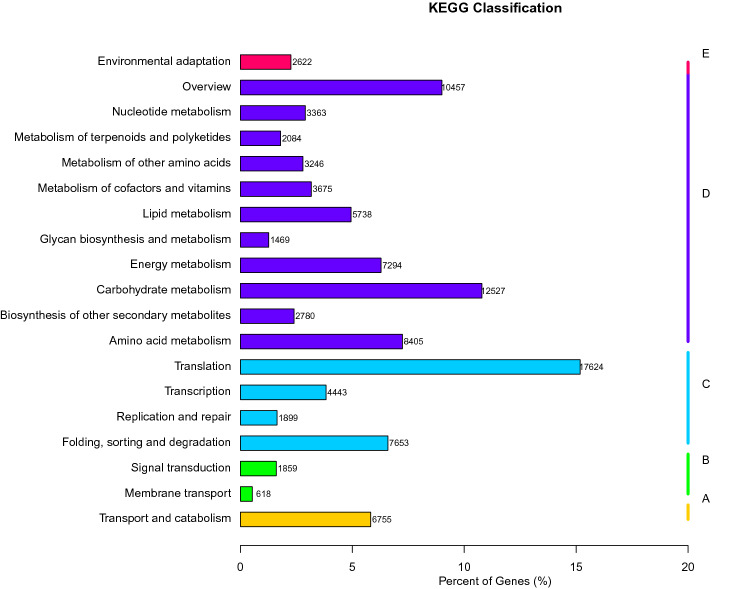
KEGG classification. The figure is divided into five branches based on the corresponding KEGG metabolic pathways: cell processes (**A**), environmental information processing (**B**), genetic information processing (**C**) information processing, metabolism (**D**) and organic systems (**E**).

The KEGG classification analysis revealed that Transport and catabolism, signal transduction, membrane transport, Translation, Carbohydrate metabolism, Energy metabolism and Environmental adaptation were among the few important pathways that were significantly enriched under Cell processes, Environmental information processing, Genetic information Processing, Information processing, Metabolism and Organic Systems of *Suaeda rigida* plant in response to salt stress (Fig. 3).

### Differential expression pattern for salt tolerance in root

**Figure 4:**
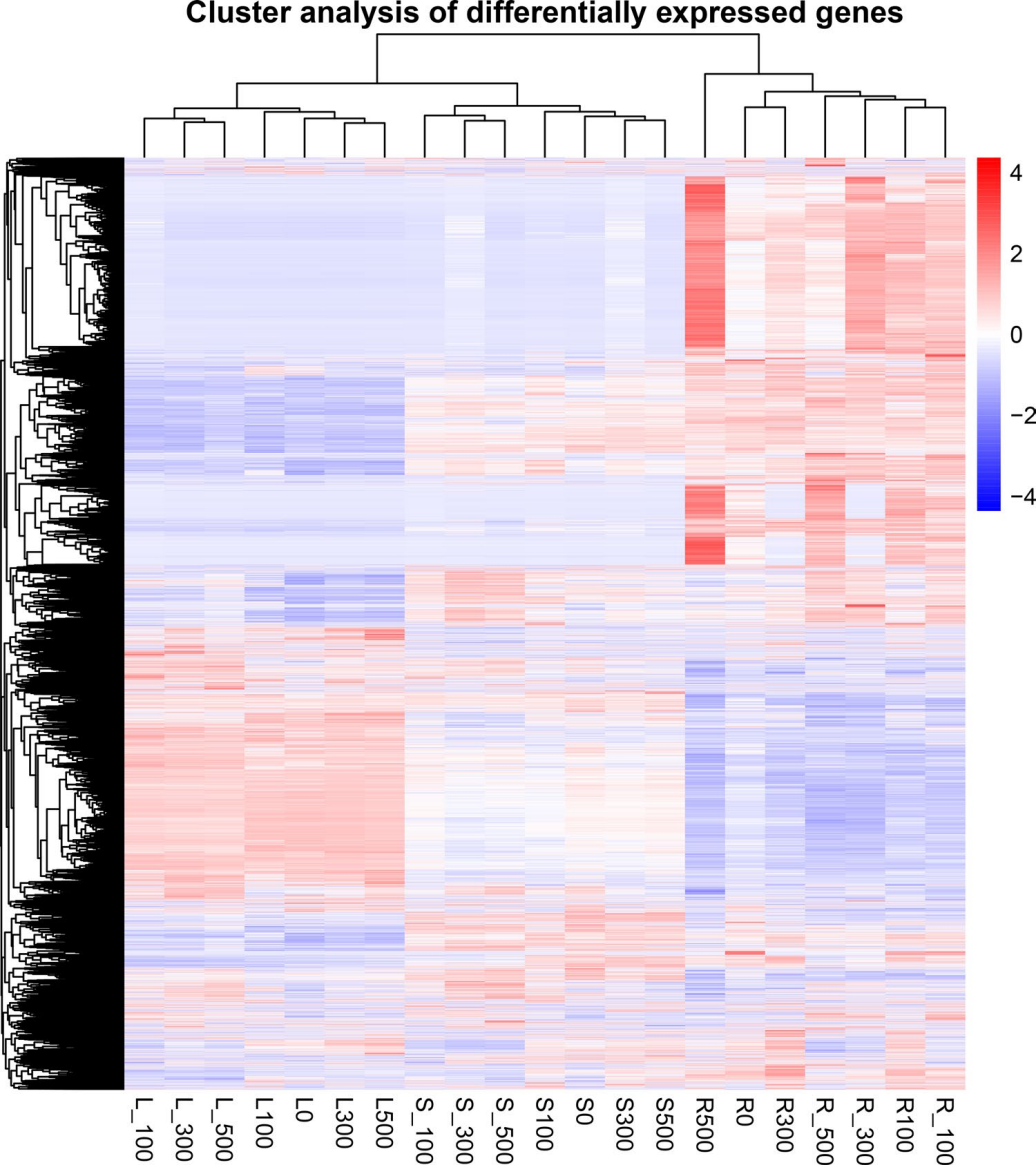
Differential gene cluster Heatmap.

Differential gene cluster analysis for all the treatments was conducted (Fig. 4). Generally, roots are the main plant tissue that are directly in contact with high salt concentration in the soil. Thus, gene expression profiles patterns in *Suaeda rigida* roots were important. Under 3 h of treatment, no up-regulated DEGs were recorded in 100 mM NaCl and 500 mM NaCl treated samples, whereas there were only 26 up-regulated DEGs in 300 mM NaCl treated samples. Meanwhile, there were 3, 37 and 0 down-regulated DEGs in 100 mM NaCl, 300 mM NaCl, 500 mM NaCl treated samples, respectively. However, in the 5 days’ treatment group, 97, 60 and 242 up-regulated DEGs were identified in 100, 300, 500 mM NaCl treated samples, respectively. Further, we identified 50, 22 and 255 down-regulated DEGs in 100, 300, 500 mM NaCl treated samples, respectively. Therefore, based on these findings, we focused on these DEGs in 5 days’ treatment group. Overall, 43 up-regulated DEGs and 24 down-regulated DEGs were present in more than two samples. For the down-regulated genes, GO biological process (BP) enrichment analysis indicated that they were enriched in nitrogen compound transport (GO: 0071705, p = 5.61E−06), organic substance transport (GO: 0071702, p = 0.010173) and intracellular protein transport (GO: 0006886, p = 0.015322) while for the up-regulated genes, they were found enriched in plant-type cell wall biogenesis related GO entries, such as plant-type cell wall biogenesis (GO: 0009832, p = 0.0088759), cell wall assembly (GO: 0070726, p = 0.0088759), extracellular matrix organization (GO: 0030198, p = 0.0088938) and plant-type cell wall organization (GO: 0009664, p = 0.027692).

### qPCR validation

**Figure 5:**
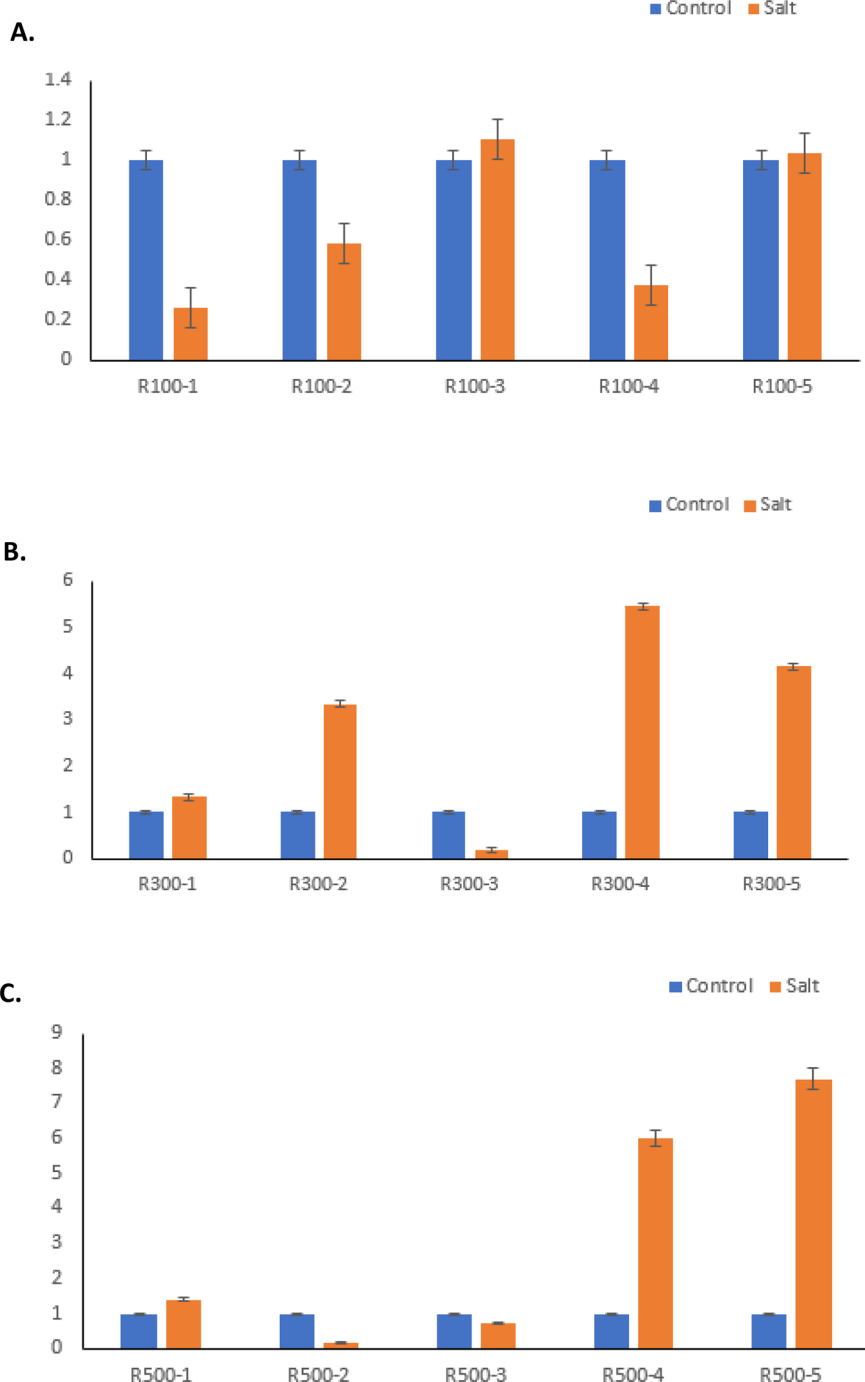
Real time-PCR validation of DE transcripts. The figure represents root treatments at three different timepoints (**A**: R100, **B**: R300 and **C**: R500). Bars represent mean ± SE (n = 3).

A set of differentially genes were randomly selected for detection and subsequent validation via qPCR, Fig. 5.

## Discussion

Herein, we conducted a transcriptomic profile analysis of *Suaeda rigida* response to saline stress conditions. In this, sequencing of the leaves, stems and roots of the plant were conducted so as to identify the salt-responsive genes. In spite of the recent development in genome sequence methods, genomic data for various non-model plants is absent. Though transcriptomic mRNA-sequencing, it has been made possible to identify and quantify transcripts even in circumstances where the reference genome sequence is not yet available^32^. Although transcriptome studies have been conducted in closely related species like *Suaeda fruticosa*, such information on *S. rigida* is still lacking.

In this study, we applied transcriptomic sequencing in identifying and analyzing the putative genes related to salt response and tolerance mechanisms in *Suaeda rigida* plants over a set gradient of NaCl treatment regimens. *Suaeda rigida* (HW Kung et GL Chu) is a lignified plant under genus *Polygonaceae*^31^. Hence, it is highly suitable in examining salt tolerant mechanisms in plants. Therefore, *Suaeda rigida* may offer a significant basis for validating salt tolerance associated genes.

In haplotypes, roots are the main plant tissue that are in direct interaction with high concentration of salt in the soil. Thus, we focused on the gene expression profiles in *Suaeda rigida* roots. From the transcriptome analysis, 28,555 DEGs were identified. A significant majority of these DEG’s clustered to four functional groups involving signal transduction, transporters, cell wall metabolism and transcription factors; that might have typical biological functions in *Suaeda rigida* salt-tolerant mechanisms.

For plants under abiotic or biotic stress, signal transduction is very crucial for adjustment in such unfavorable conditions^33–36^. From our study findings, genes associated with signal transduction processes were differentially expressed when under saline stress. Differentially expressed genes associated with control of ABA signaling pathway were highly up-regulated in 500 mM NaCl treated samples (Supplementary Data 2). The genes encoding for probable protein phosphatase 2C 25 (Cluster-47345.139659) and protein phosphatase 2C 22 (Cluster-47345.93406) were up-regulated in response to high salt levels. Phosphatase genes are considered to be important regulators of the ABA signaling in plants^37,38^. In a study conducted by Liu et al., they established that over-expression of AtPP2CG1 in Arabidopsis thaliana led to improved salt tolerance, while its depletion led to reduced salt tolerance levels^39^. Further, ZmPP2C2 over-expression in tobacco has been established to enhance tolerance to cold stress^40^. Similarly, previously conducted studies on other haplotype such as *Suaeda frutic*ose and *Suaeda glauca* demonstrated phosphatase 2C family proteins were up-regulated at high salt treatment regimens^25,41^. Thus, protein phosphatase 2C might have a crucial regulatory function in salt tolerance of *Suaeda rigida*.

Abscisic acid (ABA) is a key endogenous indicator. Abiotic factors like salinity stimulates ABA biosynthesis, which thereafter initiates the signaling pathways leading to several downstream responses^42^. Cytochrome P450 is elucidated to play a major part in ABA catabolism^43^, while other cytochrome P450s might facilitate growth and stress responses^26,27^. From our dataset, three unigenes encoding for cytochrome P450 76AD1 (Cluster-47345.95314), cytochrome P450 84A1 (Cluster-47345.105167) and cytochrome P450 CYP73A100 were up-regulated. However, the precise roles of each cytochrome P450 in *Suaeda rigida* is yet to be known. Notably, these genes might be linked to ABA signal transduction, implying ABA signal transduction may have a significant function and show prompt response of *Suaeda rigida* in the earlier stage during saline stress.

In plants, Oligopeptide transporters (OPTs) are membrane-confined proteins that have numerous transportation roles^44–46^. From our study findings, we identified one gene that encodes for an oligopeptide transporter 6 (Cluster-47345.229496), and it was up-regulated, demonstrating that it may be associated with metal transport and homeostasis during salt stress. Further, ABC proteins are actively involved in transportation of various molecules like hormones, secondary metabolites, lipids, metals and modulators of ion channels across membranes^47,48^. Thus, these ABC transporters augment drought and salt resistance^48^. Herein, three gene associated with ABC transporter families, Cluster-47345.112862, Cluster-47345.140732 and Cluster-47345.152644, were upregulated in the roots of *Suaeda rigida*. Our study findings are consistent with previously reported studies on *Suaeda glauca* and *Suaeda fruticosa*^29,41^.

As a result of saline stress, normal plant growth and development is highly affected. Cell wall modification is a response measure in plants when under biotic or abiotic stress. herein, genes related to cell wall responses were enriched. Specifically, plant-type cell wall biogenesis related GO entries, such as plant-type cell wall biogenesis (GO: 0009832), cell wall assembly (GO: 0070726), extracellular matrix organization (GO: 0030198) and plant-type cell wall organization (GO: 0009664) were up regulated in response to high salt concentration (Fig. 2). Further, genes associated with cell wall and growth were up-regulated due to salt stress. Expansin-like A2 (Cluster-47345.195511) and expansin-like B1 (Cluster-47345.119414), which are linked to cell elongation and cell wall modification^49^, were up-regulated.

Several transcription factors (TFs) take part in various significant roles in plant response mechanisms to abiotic and biotic stress. This is usually achieved through their regulation of genes associated with stress responses, such as ERFs and WRKY transcription factors^50–53^. ERFs perform various significant roles in plants’ abiotic stress tolerance by regulating stress-responsive genes^54–57^. Three genes, Cluster-47345.149382, Cluster-47345.137633 and Cluster-47345.166382, that encode for ERFs were up-regulated in *Suaeda rigida* roots under salt stress. Equally, WRKY transcription factors have been implicated in salt as well as drought tolerance in *Gossypium hirsutum*, Arabidopsis thaliana, Jatropha curcas and Jatropha curcas^58–61^. From our study findings, we identified two genes, Cluster-47345.231608 and Cluster-47345.62923, encoding WRKY’s were up-regulated under salt stress. Consistent with previously conducted studies in other plants^41,62–64^, high salinity levels led to up-regulation of bHLH transcription factor. In our study, four bHLH transcription factors, Cluster-47345.43143, Cluster-47345.120470, Cluster-47345.67446 and Cluster-47345.180519 were highly upregulated in the *Suaeda rigida* roots than in its leaves, implying they might be playing a significant role in plants’ salt tolerance and regulation in the roots.

In conclusion, 42 *Suaeda rigida* RNA-seq libraries representing various treated Sodium Chloride (NaCl) treatment regimens (100 mM NaCl, 300 mM NaCl and 500 mM NaCl), were sequenced in this study. From these sequenced libraries, a total of 2,212 M high-quality clean reads were assembled into 829,095 transcripts, from which 28,555 DEGs were identified. These DEGs contained up-regulated and down-regulated genes in *Suaeda rigida*. They comprised of genes implicated in four functional groups namely, signal transduction, transporters, cell wall metabolism and transcription factors; which might have typical biological functions in salt-tolerant mechanism of haplotype *Suaeda rigida*. These findings can be further applied in downstream genetic-based studies that aim to study the salt tolerance mechanisms and regulation in haplotype plants.

## Materials and methods

### Plant materials and RNA isolation

*Suaeda rigida* plants were grown at Tarim university, Xinjiang, China as per a previously published article of a related species^29^, but with slight adjustments. In duplicates, plants were exposed to established NaCl treatments as described in Supplementary Data 3. Sodium Chloride (NaCl) treatment regimens were 100 mM NaCl, 300 mM NaCl and 500 mM NaCl. Control group (CK) was not exposed to any treatment plan. In duplicates, the leaves (L), stem (S) and roots (R) were collected at set intervals of 3 h and 5 days post NaCl treatment (Supplementary Table 2). The collected samples were triturated to powder form in liquid nitrogen using TRIzol, total RNA was isolated using Spin Column Plant total RNA Purification Kit following the manufacturer's protocol (Sangon Biotech, Shanghai, China). The RNA quality, integrity and concentration were evaluated through the NanoPhotometer spectrophotometer (IMPLEN, CA, USA), Agilent Bioanalyzer 2100 system (Agilent Technologies, CA, USA) and Qubit RNA Assay Kit in Qubit 2.0 Flurometer (Life Technologies, CA, USA). High quality total RNA samples were utilized for downstream library preparation.

### Library preparation for Transcriptome sequencing

From each sample, 1.5 µg RNA was expended for use in library preparation. NEBNext Ultra RNA Library Prep Kit for Illumina (NEB, USA) was used to generate the sequencing libraries, as per the manufacturer’s guidelines. Index tags were added to each sample to serve as identification points. In brief, mRNA was purified from total RNA by means of poly-T oligo-attached magnetic beads. Fragmentation was done through divalent cations under high temperature in NEBNext First Strand Synthesis Reaction Buffer (5×). First strand cDNA was synthesized using random hexamer primer and M-MuLV Reverse Transcriptase (RNase H-). Next, second strand cDNA synthesis done using DNA Polymerase I and RNase H. Residual overhangs were converted into blunt ends through exonuclease/polymerase activity. After adenylation of 3′ DNA fragments ends, NEBNext Adaptor with hairpin loop structure were ligated in preparation for hybridization. Thereafter, 250 ~ 300 base pairs long cDNA fragments were selected through purification of the library segments using AMPure XP system (Beckman Coulter, Beverly, USA). Afterwards, 3 µl USER Enzyme (NEB, USA) was utilized with size-selected, adaptor-ligated cDNA at 37 °C for 15 min followed by 5 min at 95 °C before PCR. Next, PCR was conducted using Phusion High-Fidelity DNA polymerase, universal primers and Index (X) Primer. Lastly, the obtained PCR products were purified (AMPure XP system) and library quality evaluated on the Agilent Bioanalyzer 2100 system. The clustering of the index-coded samples was done on a cBot Cluster Generation System by TruSeq PE Cluster Kit v3-cBot-HS (Ilumina), as per the manufacturer’s guidelines. Thereafter, the prepared libraries were sequenced on an Illumina Hi-seq 4000 platform.

### Bioinformatics analysis

Raw fastq reads were initially assessed using trimmomatic v0.38 with parameters “PE-phred33 ILLUMINACLIP:TruSeq3-PE.fa:2:30:10 LEADING:20 TRAILING:20 HEADCROP:10 SLIDINGWINDOW:4:20 MINLEN:60”^65^. Briefly, clean reads were attained through filtering off reads with adapters, poly-*N* residuals and low-quality reads from original raw fastq reads. Simultaneously, Q20, Q30, GC-content and sequence duplication level of the clean reads were determined. For the obtained clean data, all read1 files and read2 files were pooled into single independent reads and transcriptome assembly performed using Trinity^66^, with min_kmer_cov set to 2 by default and all other parameters set as default. Gene function annotation was done using the Nr (NCBI non-redundant protein sequences); Nt (NCBI non-redundant nucleotide sequences); Pfam (Protein family); KOG/COG (Clusters of Orthologous Groups of proteins); Swiss-Prot; KO (KEGG Ortholog database)^67^; GO (Gene Ontology) databases, with an E-value threshold of 1e − 5. Picard—tools v1.41 and samtools v0.1.18 were used to sort, remove duplicated reads and merge the bam alignment results of each sample. GATK3 software^68^ was used to perform SNP calling. Raw vcf files were filtered with GATK standard filter method and other parameters (cluster: 3; WindowSize: 35; QD < 2.0 or FS > 60.0 or MQ < 40.0 or SOR > 4.0 or MQRankSum < − 12.5 or ReadPosRankSum < − 8.0 or DP < 10). SSR of the transcriptome were identified using MISA (https://pgrc.ipk-gatersleben.de/misa/misa.html), and primer for each SSR was designed using Primer3 (https://primer3.sourceforge.net/releases.php). For quantification of gene expression levels, gene expression levels were estimated by RSEM^69^ for each sample. Clean data was mapped back onto the assembled transcriptome and Read count for each gene was obtained from the mapping results. Differential expression analysis of treated versus control conditions was made via the DESeq R package (1.10.1)^70^. Benjamini and Hochberg’s filtering approach was applied on the resulting P values so as to control the false discovery rate. Genes with a P-value < 0.05 were dispensed as differentially expressed. GO enrichment analysis of the DEGs was analyzed by use of the GOseq R packages^71^, as per Wallenius non-central hyper-geometric distribution^72^. KOBAS software^73^ was utilized in evaluating the statistical enrichment of differential expression genes in KEGG pathways (https://www.genome.jp/kegg/). DEGs sequences were blast (blastx) to the genome of a closely linked species in the STRING database (https://string-db.org/); so as to obtain the predicted protein interaction (PPI) of them. Cytoscape was used to visualize these DEGs PPI^74^.

### qPCR validation

To validate the RNA-Seq data, some DEG’s were randomly selected from our constructed libraries and their levels of expression under saline conditions was assessed through qPCR analysis. In brief, 3 μg of total RNA was utilized to construct the cDNA. Reverse transcription was conducted using oligo (dT) primer as per the manufacturer’s guidelines (Fermentas, Burlington, Ontario, Canada). Primer 5 software (version 5.2.0) was utilized in designing the specific primers for the selected genes. The qPCR was conducted in a total volume of 20 μL, with each assay comprising 2 μM of the forward and reverse primers, 2 μL cDNA, and 10 μL of 2 × SYBR Green qPCR Mix (Takara, Otsu, Shiga, Japan). Thermal cycling conditions were 35 cycles of fast denaturation at 94 °C for 5 s, followed by annealing and extension at 52–55 °C for 20 s. To test the Amplicon specificity was tested through generation of a melting curve by gradually increasing the temperature to 95 °C. To establish the relative fold changes for each test, the 2 − ΔΔCT method was applied based on standardization with the reference gene. The qPCR analysis was done in triplicates, and the mean value utilized.

## Supplementary information

Supplementary Dataset 1.

Supplementary Dataset 2.

## Data Availability

All genetic data have been submitted to the NCBI Sequence Read Archive (SRA) database (https://www.ncbi.nlm.nih.gov/sra) PRJNA644132**.**
