# Supplementary material for: Transcriptomic profile analysis of the halophyte Suaeda rigida response and tolerance under NaCl stress
Source: Sci Rep. 2020 Sep 16;10:15148. doi: 10.1038/s41598-020-71529-2 (PMC7494938; doi:10.1038/s41598-020-71529-2)
Supplement: Supplementary file 1 — Supplementary Dataset 1. [file 41598_2020_71529_MOESM1_ESM.pdf]

TRANSCRIPTOMIC PROFILE ANALYSIS OF HALOPHYTE *Suaeda rigida* RESPONSE AND TOLERANCE UNDER NaCl STRESS

Zhan-Jiang Han [1,2,*], Yang Sun [1,2], Min Zhang [1,2], Jun-Tuan Zhai [1,2]

[1] College of Life Sciences, Tarim University, Alar, 843300 Xinjiang, China
[2] Xinjiang Production & Construction Corps Key Laboratory of Protection and Utilization of Biological Resources in Tarim, Alar, 843300 Xinjiang, China

Corresponding Author: hanzhanjiang126@126.com
College of Life Sciences, Tarim University, Alar, 843300 Xinjiang, China

Supplementary Table 1: Reads and reference sequence alignment list

		SAMPLE NAME	TOTAL READS	TOTAL MAPPED
CONTROL	Roots	R0_1	55500498	30071130 (54.18%)
		R0_2	55525138	31349256 (56.46%)
	Stem	S0_1	46240262	23470056 (50.76%)
		S0_2	44217816	24490234 (55.39%)
	Leaves	L0_1	45254162	26884072 (59.41%)
		L0_2	61819880	36323598 (58.76%)
100 mM NaCl 3 h	Roots	R100_1	64287884	36682532 (57.06%)
		R100_2	56376864	30834190 (54.69%)
	Stem	S100_1	51103874	27806438 (54.41%)
		S100_2	65244880	36014954 (55.20%)
	Leaves	L100_1	53719476	29942124 (55.74%)
		L100_2	45986036	26230798 (57.04%)
300 mM NaCl 3 h	Roots	R300_1	46768824	24171494 (51.68%)
		R300_2	45219284	25124564 (55.56%)
	Stem	S300_1	55102038	29355240 (53.27%)
		S300_2	48482026	26689340 (55.05%)
	Leaves	L300_1	56820286	32065088 (56.43%)
		L300_2	60589310	34840324 (57.50%)
500 mM NaCl 3 h	Roots	R500_1	43506980	28478996 (65.46%)
		R500_2	57822966	30562272 (52.85%)
	Stem	S500_1	51551058	27397054 (53.15%)
		S500_2	54642572	29860302 (54.65%)
	Leaves	L500_1	50249026	30078488 (59.86%)
		L500_2	55748250	32041454 (57.48%)
100 mM NaCl 5 d	Roots	R_100_1	56358830	30389234 (53.92%)
		R_100_2	56489744	30122410 (53.32%)
	Stem	S_100_1	50112110	28106290 (56.09%)
		S_100_2	51265754	27823224 (54.27%)
	Leaves	L_100_1	42886954	23101452 (53.87%)
		L_100_2	55390170	31450362(56.78%)
300 mM NaCl 5 d	Roots	R_300_1	47404334	26391504(55.67%)
		R_300_2	45658466	24911846(54.56%)
	Stem	S_300_1	46918936	25185682(53.68%)
		S_300_2	52979900	28481870(53.76%)
	Leaves	L_300_1	58052474	33388100(57.51%)
		L_300_2	53214490	29354866(55.16%)
500 mM NaCl 5 d	Roots	R_500_1	47150812	25988296(55.12%)
		R_500_2	44800010	24328948(54.31%)
	Stem	S_500_1	51171436	28076620(54.87%)
		S_500_2	47662434	25581126(53.67%)
	Leaves	L_500_1	59314470	33145510(55.88%)
		L_500_2	71658288	41615774(58.08%)

Sequencing output and reads mapping statistics for all 6 time points and one control were included in this table. In each condition, three tissues were used, and two biological replicates were included in each tissue.

Supplementary Table 3: Outline for the whole study experimental.

Treatment		Sample Type	Sample number
CK(Control)	Repeat I	leaf	L-0-1-3
		Stem	S-0-1-3
		Root	R-0-1-3
	Repeat II	leaf	L-0-2-3
		Stem	S-0-2-3
		Root	R-0-2-3
100 mM NaCl 3h	Repeat I	leaf	L-100-1-3
		Stem	S-100-1-3
		Root	R-100-1-3
	Repeat II	leaf	L-100-2-3
		Stem	S-100-2-3
		Root	R-100-2-3
300 mM NaCl 3h	Repeat I	leaf	L-300-1-3
		Stem	S-300-1-3
		Root	R-300-1-3
	Repeat II	leaf	L-300-2-3
		Stem	S-300-2-3
		Root	R-300-2-3
500 mM NaCl 3h	Repeat I	leaf	L-500-1-3
		Stem	S-500-1-3
		Root	R-500-1-3
	Repeat II	leaf	L-500-2-3
		Stem	S-500-2-3
		Root	R-500-2-3
		leaf	L-100-1-5D

100 mM NaCl 5d	Repeat I	Stem	S-100-1-5D
		Root	R-100-1-5D
	Repeat II	leaf	L-100-2-5D
		Stem	S-100-2-5D
		Root	R-100-2-5D
300 mM NaCl 5d	Repeat I	leaf	L-300-1-5D
		Stem	S-300-1-5D
		Root	R-300-1-5D
	Repeat II	leaf	L-300-2-5D
		Stem	S-300-2-5D
		Root	R-300-2-5D
500 mM NaCl 5d	Repeat I	leaf	L-500-1-5D
		Stem	S-500-1-5D
		Root	R-500-1-5D
	Repeat II	leaf	L-500-2-5D
		Stem	S-500-2-5D
		Root	R-500-2-5D

Sample number denotation: For example, L-0-1-3 (L is the leave, 0 is the NaCl concentration, 1 implies repetition I, 3 is the duration of sample treatment 3h). For the first letter, L represents leaf; S represents stem and R means root.
